# Biological analysis of the potential pathogenic mechanisms of Infectious COVID-19 and Guillain-Barré syndrome

**DOI:** 10.3389/fimmu.2023.1290578

**Published:** 2023-12-05

**Authors:** Hongyu Gao, Shuning Wang, Hanying Duan, Yushi Wang, Hui Zhu

**Affiliations:** Department of Neurology, The First Teaching Hospital of Jilin University, Changchun, Jilin, China

**Keywords:** COVID-19 infection, Guillain-Barré syndrome, biological analysis, SARS-CoV-2, genes

## Abstract

**Background:**

Guillain-Barré syndrome (GBS) is a medical condition characterized by the immune system of the body attacking the peripheral nerves, including those in the spinal nerve roots, peripheral nerves, and cranial nerves. It can cause limb weakness, abnormal sensations, and facial nerve paralysis. Some studies have reported clinical cases associated with the severe coronavirus disease 2019 (COVID-19) and GBS, but how COVID-19 affects GBS is unclear.

**Methods:**

We utilized bioinformatics techniques to explore the potential genetic connection between COVID-19 and GBS. Differential expression of genes (DEGs) related to COVID-19 and GBS was collected from the Gene Expression Omnibus (GEO) database. By taking the intersection, we obtained shared DEGs for COVID-19 and GBS. Subsequently, we utilized bioinformatics analysis tools to analyze common DEGs, conducting functional enrichment analysis and constructing Protein–protein interaction networks (PPI), Transcription factors (TF) -gene networks, and TF-miRNA networks. Finally, we validated our findings by constructing the Receiver Operating Characteristic (ROC) curves.

**Results:**

This study utilizes bioinformatics tools for the first time to investigate the close genetic relationship between COVID-19 and GBS. CAMP, LTF, DEFA1B, SAMD9, GBP1, DDX60, DEFA4, and OAS3 are identified as the most significant interacting genes between COVID-19 and GBS. In addition, the signaling pathway of NOD-like receptors is believed to be essential in the link between COVID-19 and GBS.

## 1 Introduction

A massive epidemic of novel coronavirus disease 2019 (COVID-19), characterized by high rates of disability and mortality, began in 2019, and the healthcare sector faces significant challenges in the current pandemic. The prevalence of COVID-19 has escalated to rank among the most significant public health well-being challenges within the category of infectious respiratory illnesses. COVID-19 can cause respiratory and neurological issues, as well as temporary loss of smell and taste. It is unclear whether the presence of neurological symptoms in individuals with COVID-19 can be attributed to either direct invasion of the virus or damage caused by immune inflammation.

Guillain-Barré syndrome (GBS) is an acute inflammatory peripheral neuropathy caused by immunity. Guillain-Barre syndrome occurs worldwide, with approximately 1 to 2 cases of Guillain-Barre syndrome in 100,000 people per year. Prior to the onset of Guillain-Barr syndrome, it has been observed that several patients have experienced prior infections, mainly upper respiratory tract infections. The emergence of the condition has been associated with different pathogenic microorganisms (1), notably Campylobacter jejuni, and Zika virus. GBS is primarily classified into two subtypes within the scholarly realm. The first is acute inflammatory demyelinating polyneuropathy (AIDP), which falls under the demyelinating category. The second subtype is acute motor axonal neuropathy (AMAN), which belongs to the axonal category. Research has indicated that the electrophysiological aspects of GBS in relation to COVID-19 typically exhibit observations that imply the presence of demyelination, aligning with Acute Inflammatory Demyelinating Polyneuropathy (AIDP) (2). The link between GBS and Severe Acute Respiratory Syndrome Coronavirus-2 (SARS-CoV-2) is strongly supported by evidence (3). However, there is still a lack of gene-level studies on both diseases. Thus, we are looking for a deeper understanding of the common molecular biology functions and pathways of COVID-19 and GBS.

**Figure 1:**
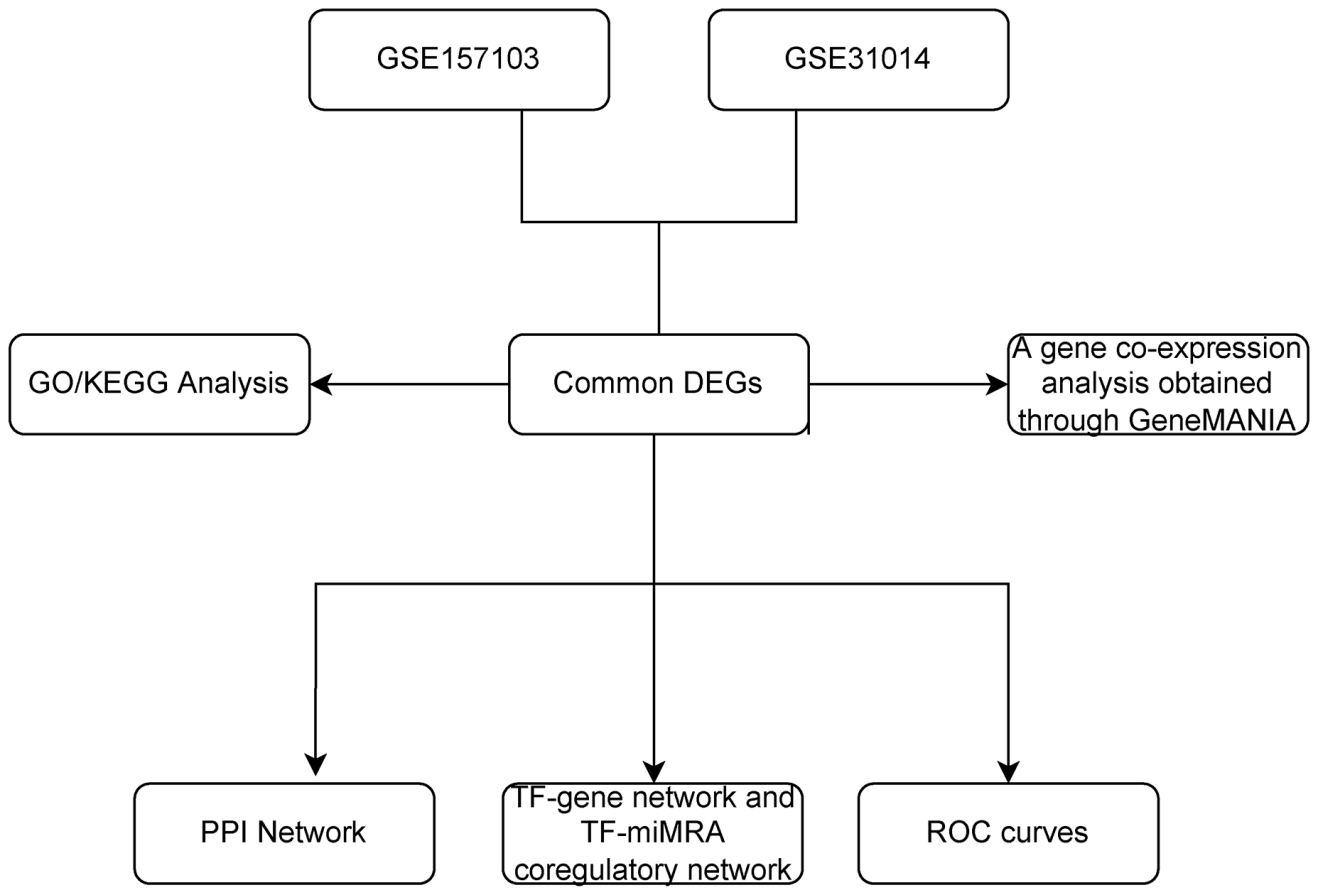
The overall workflow of research. DEGs, differentially expressed genes; GO, Gene Ontology; KEGG, Kyoto Encyclopedia of Genes and Genomes; PPI, The protein-protein interaction; TF, Transcription factor; miRNA, microRibonucleic acid.

We downloaded datasets on COVID-19 and GBS from Gene Expression Omnibus (GEO), identified differential expression of genes (DEGs) for each disease, and analyzed their enriched pathways and functions to gain insight into associated biological processes. Subsequently, to illustrate the relationships between all the genes that exhibit differential expression, a Protein–protein interaction network (PPI) network was constructed, highlighting the essential genes. Furthermore, the Transcription factors (TF)-gene regulation network and the TF-miRNA coregulation network were constructed. Finally, validation was carried out using the Receiver Operating Characteristic (ROC) curve. The primary goal of this bioinformatics study is in order to definitively establish the genetic connection between COVID-19 and GBS to gain a deeper comprehension of the pathogenesis of GBS in association with COVID-19 (4). Please refer to **Figure 1** for the flowchart.

## 2 Materials and methods

### 2.1 Dataset preparation

Microarray datasets were obtained from the Gene Expression Omnibus (GEO) database, which provides freely accessible resource for gene expression data across various diseases (http://www.ncbi.nlm.nih.gov/geo/). We selected two datasets separately: GSE157103 and GSE31014. GSE157103 consists of 102 COVID-19 samples and 26 non-COVID-19 samples obtained from Illumina NovaSeq 6000 high-throughput sequencing technology (5). The GSE31014 dataset is derived from the Affymetrix Human Genome U133A Array platform. This analysis examines the global gene expression microarray in peripheral blood leukocytes of 7 GBS patients and 7 healthy individuals (6).

### 2.2 Identification of DEGs and obtaining common DEGs

GEO2R (7), available at www.ncbi.nlm.nih.gov/geo/geo2r/, facilitates the comparison and analysis of gene expression across distinct sample groups. The DEGs of GSE157103 was analyzed with GEO2R. DEGs were assumed to be the adjusted p-value < 0.05 and | log FC | > 1.0. The identification of overlapping DEGs between the GSE157103 and GSE31014 datasets was conducted using the VennDiagram R language package (8).

### 2.3 Functional enrichment analysis

The clusterProfiler (9) package (v25.3.0) was utilized for conducting functional analysis of Gene Ontology (GO) (10, 11) and Kyoto Encyclopedia of Genes and Genomes (KEGG) pathway enrichment analysis (12) on the shared DEGs. GO or KEGG enrichment analysis are presented in bubble plot format.

### 2.4 Construction of PPI network

To gain insights into the proteins encoded by DEGs and their interactions, For the retrieval of interacting genes, we made use of the STRING database (version 11.5; https://string-db.org/cgi/input.pl) (13). A PPI network was established by implementing a confidence score threshold of 0.4 as the minimum requirement for interaction scores, while keeping all other parameters at their default settings.

### 2.5 TF gene regulatory network and TF-miRNA regulatory network

TF, which stands for transcription factors, is a group of protein molecules responsible for controlling gene expression by binding to specific sections of genes. In contrast, TF interacts with miRNAs in order to jointly control the expression of genes. In our research, we used NetworkAnalyst 3.0 to detect the TF-gene network and TF-miRNA co-regulatory network (14).

### 2.6 Construction of ROC curves for DEGs

To assess the diagnostic abilities of common genes for GBS and COVID-19, ROC curves were created and the AUC was determined using the “pROC” R package (15).

## 3 Results

### 3.1 Identifying the DEGs and shared genes between COVID-19 and GBS

**Figure 2:**
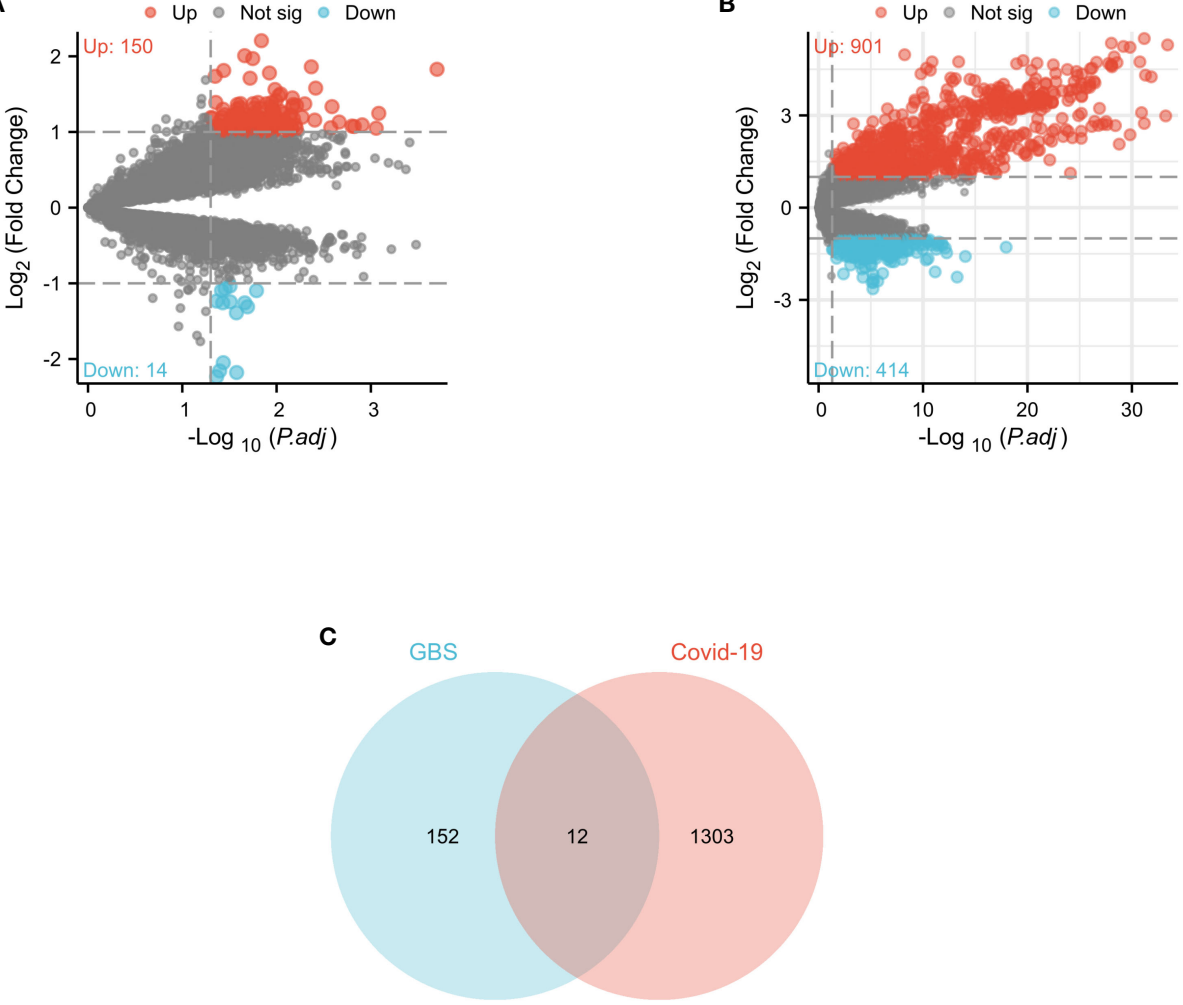
Volcano diagram and Venn diagram **(A)** The volcano map of GSE31014. **(B)** The volcano map of GSE157103. Upregulated genes are colored in red; downregulated genes are colored in blue. **(C)** The two datasets showed an overlap of 12 DEGs.

**Figure 3:**
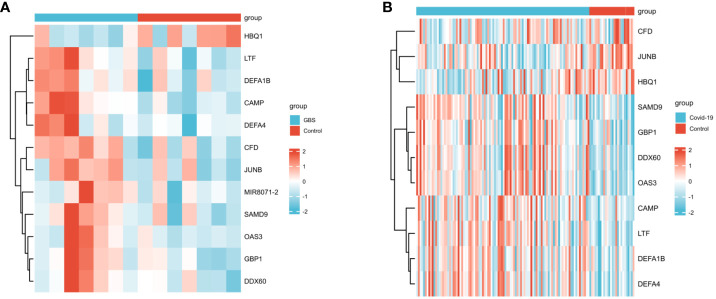
The heatmap of two datasets **(A)**The heatmap showed the expression of 12 DEGs in GSE31014; **(B)** The heatmap showed the expression of shared DEGs in GSE157103.

In the GSE31014 dataset, the sum of 164 DEGs was detected, with 150 genes up-regulated and 14 genes down-regulated (**Figure 2A**). In the GSE157103 dataset, a comprehensive analysis revealed the identification of 1315 DEGs. 901 genes exhibited up-regulated, while 414 genes displayed down-regulated (**Figure 2B**). We identified 12 common DEGs: HBQ1, CAMP, LTF, CFD, DEFA1B, SAMD9, GBP1, JUNB, DDX60, MIR8071-2, DEFA4, and OAS3, as shown in the Venn diagram (**Figure 2C**). Interestingly, among the 12 shared DEGs, only HBQ1 was found to be down-regulated in the disease group in the GSE31014 dataset, while the expression of the remaining genes was up-regulated (**Figure 3A**). In dataset GSE157103, the disease group exhibited lower expression levels of HBQ1, CFD, and JUNB genes compared to the control group, while the expression levels of other genes were higher (**Figure 3B**).

### 3.2 GO and KEGG enrichment analysis for DEGs

**Figure 4:**
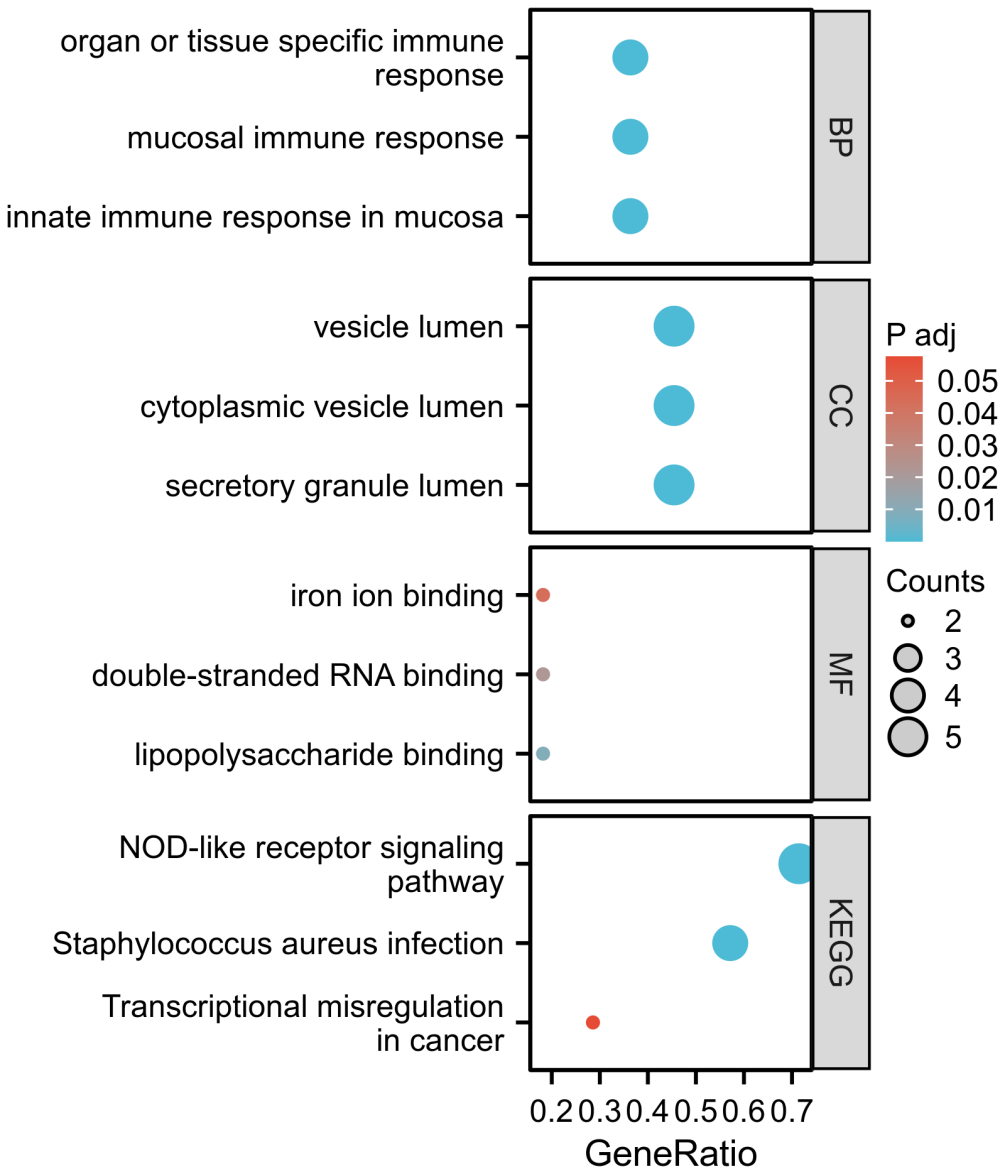
The GO and KEGG functional enrichment analysis.

GO enrichment analysis revealed significantly enriched pathways (p-value<;0.05) in various categories such as biological processes (BP), cell composition (CC), and molecular functions (MF). Based on the analysis results of GO enrichment, biological processes was focused on: innate immune response in mucosa, organ or tissue-specific immune response, and mucosal immune response. The DEGs including CAMP, LTF, DEFA1B, and DEFA4 were found to be enriched in the biological processes mentioned above. Cell composition was found to be higher in the vesicle lumen, cytoplasmic vesicle lumen, and secretory granule lumen. The genes CAMP, LTF, DEFA1B, DEFA4, and CFD play crucial roles in the cell composition mentioned above. Regarding molecular function, double-stranded RNA binding, lipopolysaccharide binding, and iron ion binding are among the highest rankings. The KEGG pathway enrichment analysis was conducted to ascertain the shared pathways between 12 common DEGs. The top 3 KEGG human pathways are the NOD-like receptor signaling pathway, Staphylococcus aureus infection, and Transcriptional misregulation in cancer (**Figure 4**). The DEGs involved in the NLR signaling pathway in this study were CAMP, DEFA1B, GBP1, DEFA4, and OAS3.

### 3.3 PPI network construction

**Figure 5:**
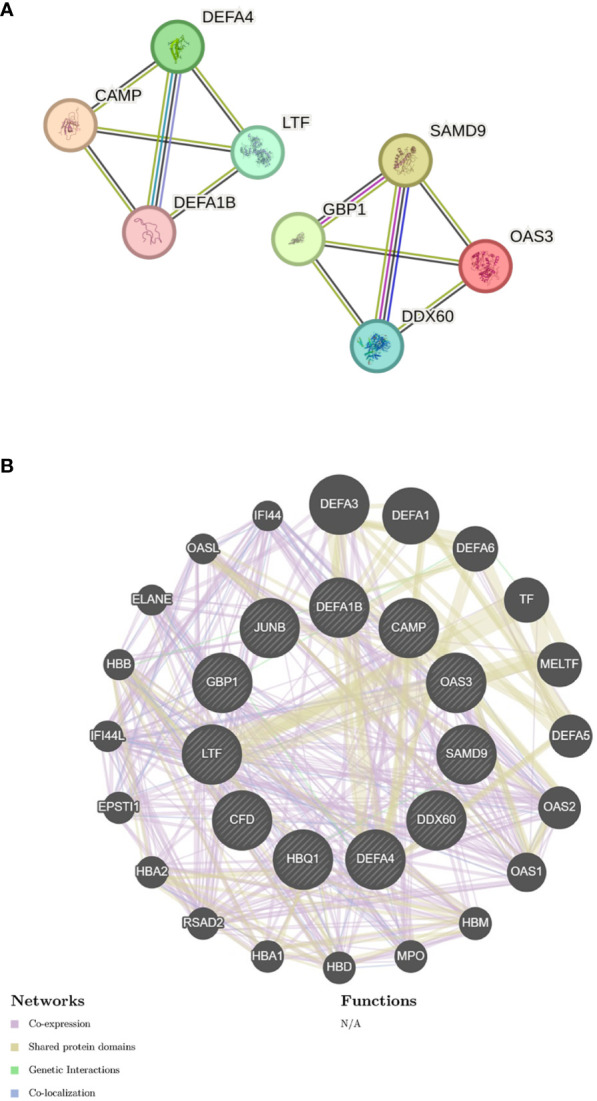
Gene network analysis **(A)** PPI network of common DEGs. **(B)** shared DEGs were analyzed via GeneMANIA.

I submitted the 12 DEGs to the STRING database and utilized the generated data to create a visual representation. The PPI network for the shared genes, which consisted of 11 nodes and 12 edges (**Figure 5A**). We used the GeneMANIA database to create a detailed network of gene interactions. This helps us understand the biological functions of the genes that were expressed differently. The results show that 76.91% of genes are Co-expression, 17.39% are Shared protein domains (**Figure 5B**). Please refer to **Supplementary Material** for more details.

### 3.4 TF-gene network and TF-miRNA coregulatory network

**Figure 6:**
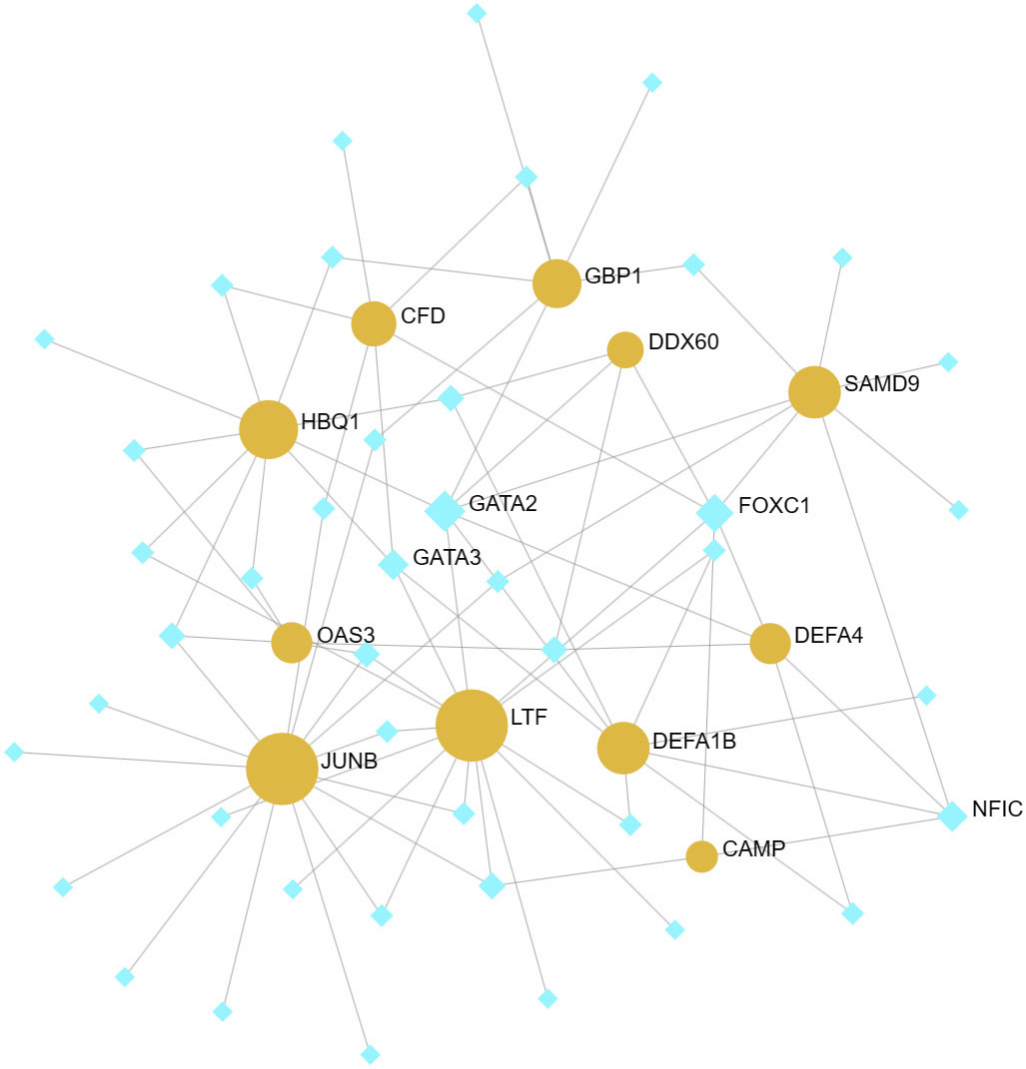
TF-gene networks. This network consists of 11 Seeds, 54 Nodes, and 86 Edges. The highlighted orange nodes represent shared DEGs, while the remaining blue nodes represent TFs.

**Figure 7:**
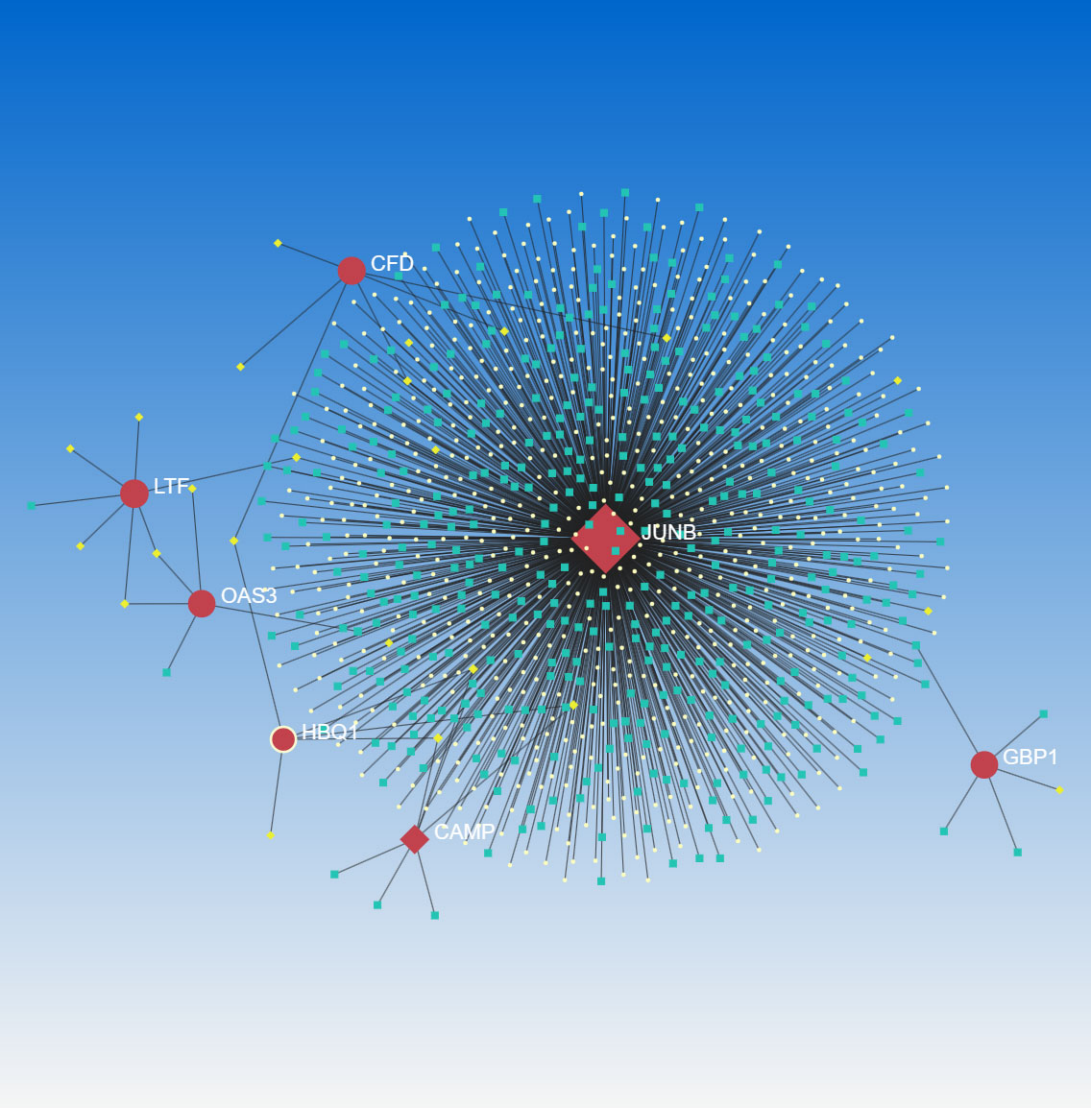
TF-miRNA coregulatory network. The nodes in red color are the DEGs, a yellow node represents TFs and other nodes indicate miRNAs. This network comprised 9 Seeds, 1100 Nodes, and 1109 Edges.

The identification of common DEGs in the TF-gene network and the TF-miRNA co-regulatory network was carried out using NetworkAnalyst 3.0. This network consists of 11 Seeds, 54 Nodes, and 86 Edges (**Figure 6**). Within the network of TF-gene interactions, LTF, JUNB and HBQ1 demonstrate numerous connections with other TFs. FOXC1 and GATA2 are the most active transcription factors in TF-gene interactions. Afterward, we constructed a TF-miRNA Coregulatory Network using the same approach, aiming to predict the interactions among shared DEGs, TFs and miRNAs (**Figure 7**). This network comprised 9 Seeds, 1100 Nodes, and 1109 Edges. The gene that is most closely associated with TFs and miRNAs is JUNB.

### 3.5 Construction of ROC curves for shared DEGs

**Figure 8:**
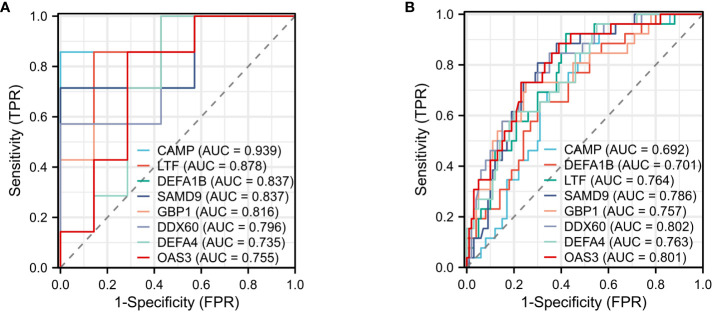
ROC curve **(A)** ROC curve for diagnostic validity validation in GSE31014. **(B)** ROC curve for diagnostic validity validation in GSE157103.

The effectiveness of the 12 common DEGs was evaluated through the construction of ROC curves in the GBS dataset to determine their diagnostic efficacy. CAMP (AUC: 0.939), LTF (AUC: 0.878), DEFA1B (AUC: 0.837), SAMD9(AUC: 0.837), GBP1(AUC: 0.816), DDX60 (AUC: 0.796), DEFA4 (AUC: 0.735), and OAS3(AUC: 0.755) were found to be useful for distinguishing GBS patients from healthy individuals showed good diagnostic efficiency (**Figure 8A**). The COVID-19 dataset revealed CAMP (AUC: 0.692), LTF (AUC: 0.764), DEFA1B (AUC: 0.701), SAMD9(AUC: 0.786), GBP1(AUC: 0.757), DDX60 (AUC: 0.802), DEFA4 (AUC: 0.763), and OAS3(AUC: 0.801) that exhibited superior diagnostic capabilities (**Figure 8B**).

## 4 Discussion

GBS refers to a collection of autoimmune peripheral neuropathies that are typically acquired following bacterial or viral infections. According to reports, there is a close association between GBS and various viruses such as Cytomegalovirus (CMV), Epstein-Barr virus (EBV), Zika virus, and West Nile virus. However, there is limited research on the connection between COVID-19 and GBS. Therefore, our objective was to investigate the molecular biological mechanisms and pathways that are common to both COVID-19 and GBS, as well as to establish the correlation between them.

In conducting this research, two separate gene microarray databases for COVID-19 and GBS were analyzed using bioinformatics methods. Through GEO database, we have successfully identified a total of 12 common DEGs distinguishing COVID-19 from GBS. Based on the GO enrichment analysis, it was observed that the DEGs were primarily enriched in signal pathways that regulate immune response. Furthermore, the KEGG enrichment analysis of DEGs primarily focused on NOD-like receptor signaling pathways.

### 4.1 How does the signaling pathway function?

The innate immune system acts as the main protective mechanism against invading pathogens. It relies on pattern recognition receptors (PRRs) to detect pathogen-associated molecular patterns (PAMPs) and trigger signaling pathways that lead to the elimination of the pathogens. There are two pathways for signaling: NOD-like receptor (NLR) and Toll-like receptor (TLR) pathways (16). The NLR pathway includes a protein family that acts as PRRs (17). It can be involved in the formation of inflammatory vesicles, where the role of NLR thermal protein domain associated protein 3 (NLRP3) inflammatory vesicles is crucial in the host antiviral immune response. This complex is made up of NOD-like receptor NLRP3, articulator ASC, and cystein-1 (18). Activating NLRP3 inflammasome causes lysosomal damage, mitochondrial dysfunction, and metabolic changes, and triggers cell pyroptosis. This process is a programmed cell death pathway that depletes T lymphocytes and mediates immune cell depletion. Ultimately, it can also result in a cytokine storm, leading to acute lung injury, acute respiratory distress syndrome, and systemic inflammatory response syndrome. In the pathogenesis of neurological diseases, NLRP3 inflammatory vesicle activation occurs mainly in microglia and macrophages. Activation leads to the release of active cystathione-1 further promoting Interleukin (IL)-1 and IL-18 maturation. Cytokines can exacerbate neuroinflammation and ultimately lead to neuronal cell death. Neurological disorders such as Alzheimer’s disease, Parkinson’s disease, traumatic brain injury, stroke, depression, and multiple sclerosis are often associated with the progression of NLRP3 inflammatory vesicles. However, there have been fewer studies conducted on the association between GBS and these vesicles (19).

The TLR signaling pathway involves a group of receptors called Toll-like receptors (TLRs) (20). These receptors identify molecules released by pathogens and trigger the innate immune system. Numerous hypotheses have been put forth to elucidate the pathogenesis of GBS, encompassing the potential role of TLR. TLR ligands are composed of endogenous and exogenous ligands. Each isoform is capable of signaling after recognizing a specific ligand. TLR2 is responsible for the recognition of peptidoglycan (PGN), whereas TLR4 is responsible for the recognition of lipopolysaccharide (LPS). NF-B levels can be increased through MyD88-dependent or non-dependent pathways, resulting in the secretion of pro-inflammatory cytokines. Additionally, Studies have demonstrated that it has the capacity to boost the generation of certain cytokines, including IL-1, IL-6, IL-12, tumor necrosis factor (TNF), and interferon (IFN) (21). These cytokines are believed to contribute significantly to the development of GBS (22). Moreover, TLR plays a crucial role in promoting the development and maturation of immune cells, which may have implications in the pathogenesis of GBS (20, 23, 24).

TLR2 is capable of detecting β-coronavirus infection through recognition of E protein, resulting in TNF-α, IFN-γ and other inflammatory cytokines. The E protein is a vital component of coronaviruses, serving as a structural protein with ion channel capabilities. This mechanism has the ability to form pores consisting of proteins and lipids in the membrane, which aids in the transportation of ions and initiates the formation of the NLRP3 inflammasome (25). The activation of NLRP3 triggers the secretion of the pro-inflammatory cytokines IL-1β and IL-18 as a result of β coronavirus infection. Inflammatory response induced by β-coronaviruses involving TLR2 relies on the presence of Myd88, which acts as a TLR adaptor protein (26). Inhibitors or antibodies that target TLR2 can be utilized to block the release of harmful cytokines and chemokines, which can help prevent cytokine storms. This approach could potentially serve as a treatment option for COVID-19-related GBS and offer valuable insights for managing the disease (26). Our analysis suggests that the NLR and TLR signaling pathways may be key in the link between COVID-19 and GBS. These pathways could provide new targets for treating COVID-19-related GBS.

### 4.2 How do genes exert their influence?

According to GO enrichment analysis, the genes CAMP, LTF, DEFA1B, and DEFA4 play vital roles in innate immune response in mucosa. Antimicrobial peptides (AMPs) play a vital role in the innate immune response against pathogens, such as viruses, by defending the body against invading microorganisms. The defensins (α-defensins and β-defensins) and the cathelicidin LL-37 are the most extensively studied classes of AMPs in humans (27). The CAMP gene, also known as LL-37, is located on chromosome 3p21.31. The CAMP protein is composed of 37 amino acids in a spiral shape and a type of Cathelicidin antimicrobial peptide (28). Recent studies indicate that LL-37 is a host defense peptide with direct anti-SARS-CoV-2 activity. Some researchers have proposed that CAMP may exert its anti-COVID-19 infection effects through several mechanisms. On one hand, the activation of the vitamin D pathway through TLR receptors induces the expression of CAMP genes. On the other hand, it can directly inhibit the replication and transmission of SARS-CoV-2. Additionally, LL-37 plays a crucial role in neutrophil extracellular traps (NETs) (29).

In this study, we have observed significantly elevated levels of α-defensins (DEFA1 and DEFA3) in COVID-19 and GBS. These biomarkers may be shared by two diseases. Extensive evidence suggests that α-defensins play a significant role in the innate immune response originating from neutrophils and monocytes (30). Recently some studies indicate that elevated Levels of Alpha-Defensins (DEFA1) is associated with disease severity in COVID-19 and can inhibit SARS-CoV-2 infection (31–33). Lactoferrin (LTF), a glycoprotein with versatile iron-binding capabilities, holds significant importance in immune regulation and defense mechanisms against bacteria, fungi, and viruses. It has been studied against a broad range of viruses, including SARS-CoV-2. LTF prevents viral entry by binding to both cell surface molecules and viral particles. In addition, it can suppress virus replication (34).

The DEGs involved in the NLR signaling pathway in this study were CAMP, DEFA1B, GBP1, DEFA4, and OAS3. While CAMP and OAS3 were involved in the gamma-type interferon-mediated signaling pathway. Studies have demonstrated that SARS-CoV-2 ORF6 functions as an immune evasion tactic by obstructing STAT1’s nuclear translocation, which counteracts type II IFN-mediated immune responses of the host, resulting in an increase in transcriptional expression of NLRC5 and a decline in CITA activity (21). The OAS gene family plays a crucial role in innate immunity and the execution of antiviral biological processes. It is an interferon-induced dsRNA-activated antiviral enzyme. ATP activation leads to the formation of adenosine oligomers that activate RNA enzyme L, which then degrades the cell (10). The OAS/RNase L pathway assumes a critical function in the establishment of an antiviral state and the prevention of viral infection (35, 36). According to prior research, it has been found that OAS cluster variants are linked to a heightened susceptibility to severe COVID-19. Additionally, the OAS gene family has been identified as a crucial component in the innate antiviral mechanism related to SARS-CoV-2 infection. Numerous studies indicate that IFN-γ has a significant pro-inflammatory role in the development of GBS (37).

DDX60 is a cytoplasmic helicase induced by IFN. This substance activates the RIG-I pathway and facilitates the production of type I IFN. It serves as an upstream factor for RIG-I. In the event of viral infection, phosphorylation of DDX60 can be induced by epidermal growth factor receptor (EGFR), reducing antiviral activity (38). The M protein from SARS-CoV-2 weakens the body’s antiviral response and increases viral replication by hindering the production of type I and type III IFN through targeting the RIG-I/MDA-5 signaling (39). It has been suggested that DDX60 may interact directly with SARS-CoV-2 viral proteins and is considered a key host factor during SARS-CoV-2 infection. No known link exists between the genes DDX60 and GBS, though they may be related to autoimmune conditions (40).

### 4.3 What are the associations between TFs and microRNAs with two diseases?

After careful selection, it has been determined that FOXC1 and GATA2 are the most significant transcription factors in the TF-gene network. In previous bioinformatics analyses, numerous researchers have observed that among the identified TFs, FOXC1 and GATA2 have been established as crucial regulatory factors in COVID-19 (41–43). Their association with GBS is relatively limited. In TF-miRNA coregulatory network, JUNB is a transcription factor that plays a role in the regulation of gene activity after the initial growth factor response.

### 4.4 What are the achievements and shortcomings of this research study?

This study explores, for the first time, the close genetic relationship between COVID-19 and GBS using bioinformatics tools. CAMP, LTF, DEFA1B, SAMD9, GBP1, DDX60, DEFA4, OAS3 are identified as the most important interacting genes between COVID-19 and GBS. The KEGG enrichment analysis of key genes suggests that the NLR signaling pathway is a critical pathway in COVID-19-related GBS. In our study, we aim to shed light on the significant contribution of the NLR signaling pathway to the development of GBS associated with COVID-19. Our study, however, has a few limitations that should be acknowledged. Firstly, we utilized only one dataset for the analysis of each disease. Of course, in the future, we will consider utilizing additional datasets for more in-depth analysis. In addition, it is essential to conduct external validation in order to authenticate our findings. In order to complete our work, we need to validate the function of the hub gene in an *in vitro* model. This will be our primary focus in future efforts.

## Data availability statement

Information for existing publicly accessible datasets is contained within the article.

## Author contributions

HG: Writing – original draft, Writing – review & editing. YW: Writing – review & editing. HD: Writing – review & editing. SW: Writing – review & editing. HZ: Writing – original draft.
